# Spontaneous detachment of *Streptococcus mutans* biofilm by synergistic effect between zwitterion and sugar alcohol

**DOI:** 10.1038/s41598-017-08558-x

**Published:** 2017-08-14

**Authors:** Jong Hyun Lim, Sang-Hun Song, Hyun-Sub Park, Jeong Rae Lee, Sang-Min Lee

**Affiliations:** R&D Campus, LG Household & Health Care, Yuseong-gu, Daejeon 34114 Republic of Korea

## Abstract

A biofilm, a community of microorganisms, is highly resistant to antibiotics, resulting in massive losses in various areas. We herein present a strategy to remove *Streptococcus mutans* biofilms through a spontaneous exfoliation by the synergistic effect between zwitterion and sugar alcohols. It is assumed that the anionic site of zwitterion can be coupled with sugar alcohols and the cationic site remains in the state of lacking electrons. The cationic site allows the complexes to be delivered to negatively charged exopolysaccharides of biofilms. This strategy facilitates a significant increase in the ability of sugar alcohols to disperse aggregated exopolysaccharides. In this work, it was demonstrated that the mixture of betaine and erythritol existed as a complex in water and that the complex induced a spontaneous detachment of biofilms from the surface to which the biofilms had been adhered. This detachment resulted from a reduction in adhesive forces of the biofilms due to an increase in solubility of bacterial exopolysaccharides. The effects triggered by the formation of complex between zwitterion and sugar alcohol provide a simple and safe way to remove biofilms without antibiotics and physical forces.

## Introduction

Microorganisms exist in the form of biofilm to protect themselves in hostile environments. Biofilms are surrounded by extracellular polymeric substances (EPS) that inhibit penetration of external molecules^1^. Thus, the biofilms are up to 1,000 times more resistant to antibiotics than their planktonic counterparts^2^. EPS are also involved in bacterial adherence and prevent detachment of biofilms^3^. The formation of biofilms results in massive losses in a variety of areas including food, water-treatment, paper, and medical industries^4, 5^. In particular, biofilms formed in the human body cause a lot of health problems such as rhinosinusitis^6^, otitis media^7^, cholesteatoma^8^ and various infections^9^.

Dental plaque is a representative biofilm in the human body, and causes periodontal diseases such as gingivitis, periodontitis and dental caries^10–12^. Many researches have been performed to inhibit the formation of dental biofilms^13–16^ and to remove mature biofilms^17–20^. The major obstacle to inhibit or remove dental plaque is susceptibility to irritation in oral mucosa^21^, especially, in non-keratinized epithelium of sublingual and buccal regions^22^. The non-keratinized epithelium is considerably more sensitive and permeable to external molecules than normal keratinized epithelium^23, 24^. Moreover, ingesting substances in the oral cavity is inevitable. Hence, the safety of substances exposed to the oral environment must be ensured. For these reasons, most of beneficial agents including natural extracts^25, 26^ and enzymes^27, 28^, which are generally regarded as safe, are not readily available in practice despite their excellent efficacy to inhibit or remove biofilms.

Erythritol, a natural sugar alcohol, is recently receiving a great deal of attention as a solution to the problems of dental biofilms. It has a sweetness of 60–80% compared to sucrose, but it provides no calories and does not affect blood glucose and insulin levels^29^. Many publications have proven very low toxicity of erythritol by ingestion^29–32^. Interestingly, recent studies have confirmed the inhibitory effect of erythritol on biofilm formation^33–38^. Clinical studies have also shown that erythritol inhibited the formation of dental plaque^39, 40^. This inhibition resulted from the suppression of bacterial growth^36^ and the decrease in expression of endopeptidase^37^, glucosyltransferase and fructosyltransferase genes^38^. Erythritol also enhances the penetration of antibiotics into mature biofilms^41, 42^. The action of erythritol on this penetration-enhancing effect is unclear. Ichikawa *et al*. were speculated that erythritol diffused into the biofilm and this diffusion weakened the cohesion of biofilms^41^. The effect of erythritol, which inhibits the formation of biofilms and enhances the penetration of antibiotics, is superior to those of other sugar alcohols such as xylitol, which is widely used for oral health in our daily lives^43^. However, erythritol, like other sugar alcohols, has the drawback that it is effective only at relatively high concentrations (several percent by weight). Also, continuous proposals for influences on mature biofilms are required to broaden the scope of applications.

We herein present a strategy to dramatically increase the effect of erythritol on weakening the cohesion of mature biofilms. This strategy began with the formation of a complex composed of erythritol and zwitterionic molecule. We hypothesized that an anionic part of zwitterion interacts with hydroxyl groups of erythritol to form a complex by intermolecular interactions and a cationic part can act to transfer the complex to negatively charged EPS. Upon contact with the complex, exopolysaccharides of biofilms were dispersed, and mature biofilms were consequently detached from the surface without physical forces. This article provides the effect of the complex composed of zwitterion and sugar alcohol on *S. mutans* biofilms.

## Results

### Intermolecular interactions between betaine and erythritol

In this study, betaine, a safe food ingredient and nutritional supplement^44^, was used as a zwitterion. Intermolecular interactions between betaine and erythritol with a molar ratio of 2:1 in water were first identified using Fourier-transform infrared (FT-IR) spectroscopy. The FT-IR spectra of betaine, erythritol and the mixture thereof in water were measured. Betaine and erythritol were dissolved in D_2_O at a concentration of approximately 10% by weight. D_2_O was used rather than H_2_O to clearly identify the shift of OH-vibrational state. The most prominent change in the spectrum of the mixture is a blue-shift of the peak derived from asymmetric stretching vibration of carboxylate in betaine. The peak at 1612 cm^−1^ was shifted to 1620 cm^−1^ as shown in Fig. S1a and c when betaine and erythritol were dissolved together in water. The shift is presumably due to newly formed hydrogen bonding between betaine and erythritol. A more detailed description is given in the Supplementary Information.

To further confirm the intermolecular interaction between the betaine-erythritol mixture and water, a 2D nuclear magnetic resonance (NMR) spectrum of the mixture composed of betaine, erythritol and water at a molar ratio of 2:1:3 was acquired. For the NMR analysis, the mixture of betaine, erythritol and water was completely dissolved in dimethyl sulfoxide (DMSO)-*d*6, and a final concentration of the mixture was approximately 1% by weight. The NMR spectrum was recorded with nuclear Overhauser enhancement spectroscopy (NOESY)^45^. The NMR spectrum in Fig. S2 indicates the presence of interactions between hydroxyl groups of erythritol and H_2_O. Furthermore, alpha protons of betaine and hydroxyl groups of erythritol were in close proximity, which obviously indicates that betaine and erythritol were combined into a complex even though they were dissolved in H_2_O and DMSO.

### Detachment of biofilm by betaine-erythritol mixture

**Figure 1:**
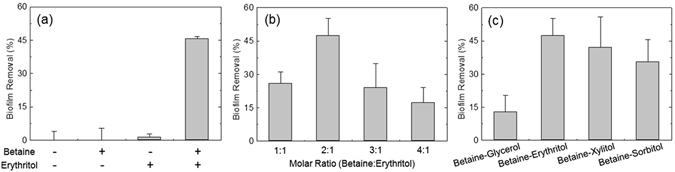
(**a**) Biofilm-removal efficiency of a mixture consisting of betaine and erythritol. *S. mutans* biofilms were exposed to 5.6 mM betaine, 2.8 mM erythritol and a mixture thereof, respectively. (**b**) Effect of the molar ratio between betaine and erythritol on biofilm removal. Weight fractions of the mixtures of betaine and erythritol were 0.1% in all samples. (**c**) Effect of hydrogen bond donor (HBD) combined with betaine on biofilm-removal efficiency. Betaine was combined with glycerol, erythritol, xylitol, and sorbitol at molar ratios of 3:2, 2:1, 5:2, and 3:1, respectively. In all figures, each value and error bar indicates mean and standard deviation (SD), respectively (*n* = 3–6).

The mixture of betaine and erythritol induced the detachment of *S. mutans* biofilms. The efficiency of biofilm detachment was assessed *via* Alamar blue assay on microplates^46^. The biofilms established on microplates were immersed in sample solutions for 10 min without any physical forces such as shaking and pipetting. Then, the solutions were gently removed. After this process, the amount of residual biofilm was quantitatively analyzed by Alamar blue assay, a method to determine the amount of live bacteria using an oxidation-reduction indicator. As shown in Fig. 1a, solutions containing erythritol and betaine, respectively, did not induce the detachment of biofilms, whereas a solution containing them resulted in the significant detachment. In this experiment, aqueous solutions containing 5.6 mM betaine, 2.8 mM erythritol and the mixture thereof were used.

Molar ratio between betaine and erythritol affected the efficacy of the mixture to induce the detachment of biofilms. In order to examine the effect of molar ratio, four samples were prepared. The samples had different molar ratios between betaine and erythritol, but weight fractions of mixtures of two substances were equal to 0.1% in all samples. When biofilms were immersed in a solution containing betaine and erythritol at a ratio of 2:1, the largest amount of biofilms was detached (Fig. 1b).

Hydrogen bond donor (HBD) coupled with betaine also influenced the efficacy of the mixtures. The composition of betaine and sugar alcohols was chosen to be the most efficient for biofilm removal. Aqueous solutions containing betaine-glycerol, -erythritol, -xylitol, and -sorbitol with molar ratios of 3:2, 2:1, 5:2 and 3:1, respectively, were prepared. Betaine and sugar alcohols were mixed, and then diluted with distilled water (DW). All sample solutions contained the mixtures of betaine and sugar alcohols at a concentration of 0.1% by weight. And an increase in viscosity of the solutions due to the addition of the mixtures was negligible. Among these solutions, the solution containing betaine and erythritol removed the largest amount of biofilms (Fig. 1c).

**Figure 2:**
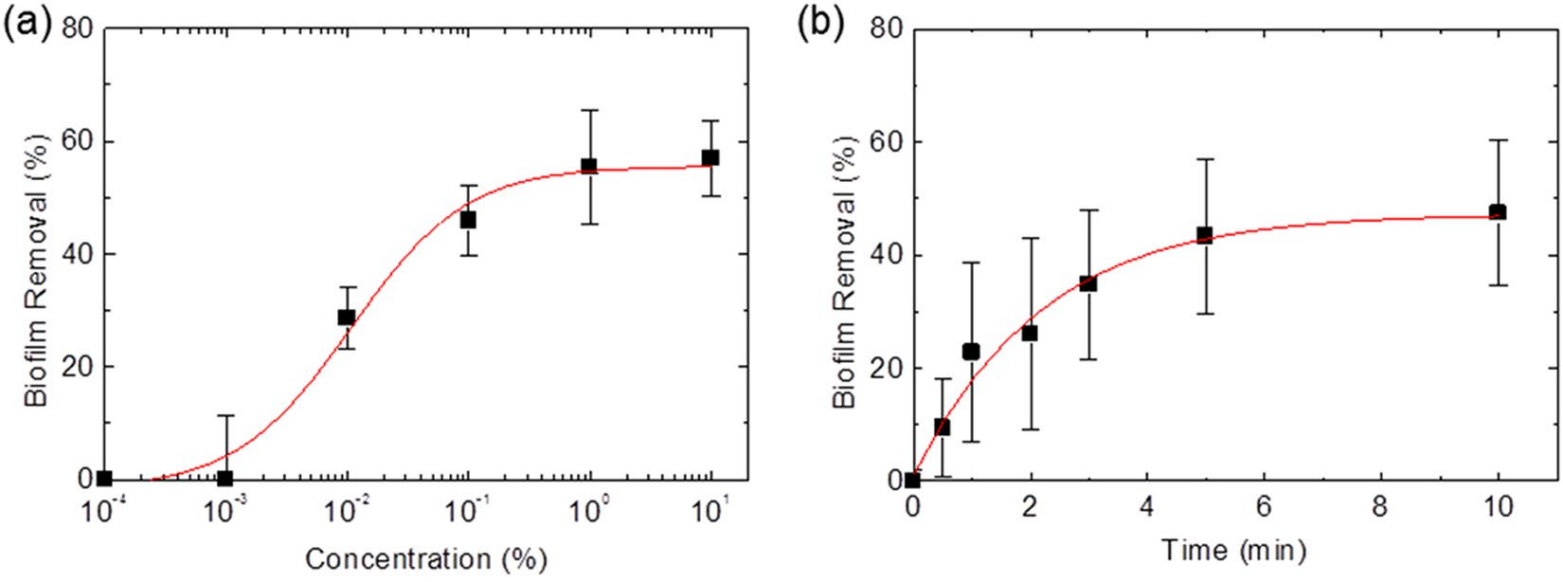
(**a**) Dose-dependent correlation between biofilm-removal efficiency and the amount of betaine-erythritol mixture. Concentration value represents the weight fraction of the mixture of betaine and erythritol. A mixture of betaine and erythritol at a 2:1 molar ratio was serially diluted with water. (**b**) Time-dependent correlation between biofilm-removal efficiency and exposure time of the solution containing betaine and erythritol at a molar ratio of 2:1. The solution used included 0.1% betaine-erythritol mixture, i.e., 5.6 mM betaine and 2.8 mM erythritol. Each point and error bar indicates mean and SD, respectively (*n* = 3–6).

The mixture of betaine and erythritol induced the detachment of biofilms even when diluted to low concentrations in water. Betaine and erythritol were mixed at a molar ratio of 2:1, and then the mixture was serially diluted with DW. In Fig. 2a, the concentration value on the x-axis represents the weight fraction of the mixture of betaine and erythritol in the total weight of solution. The efficiency of biofilm detachment increased with the concentration of the mixture, reaching a plateau at around 1%.

The time required for biofilm detachment was investigated. In this experiment, 0.1% betaine-erythritol solution was used. When the weight fraction of the mixture in water is 0.1%, 5.6 mM betaine and 2.8 mM erythritol are contained in water. Therefore, the molar ratio of betaine and erythritol contained in the solution was 2:1. *S. mutans* biofilms were immersed in the sample solution for different periods of time, and the amount of biofilm detached was assessed. As shown in Fig. 2b, the amount of biofilm detached increased with contact time with the mixture. Although approximately five minutes was required for the maximum level of detachment, higher than 70% of efficacy was achieved within three minutes.

**Figure 3:**
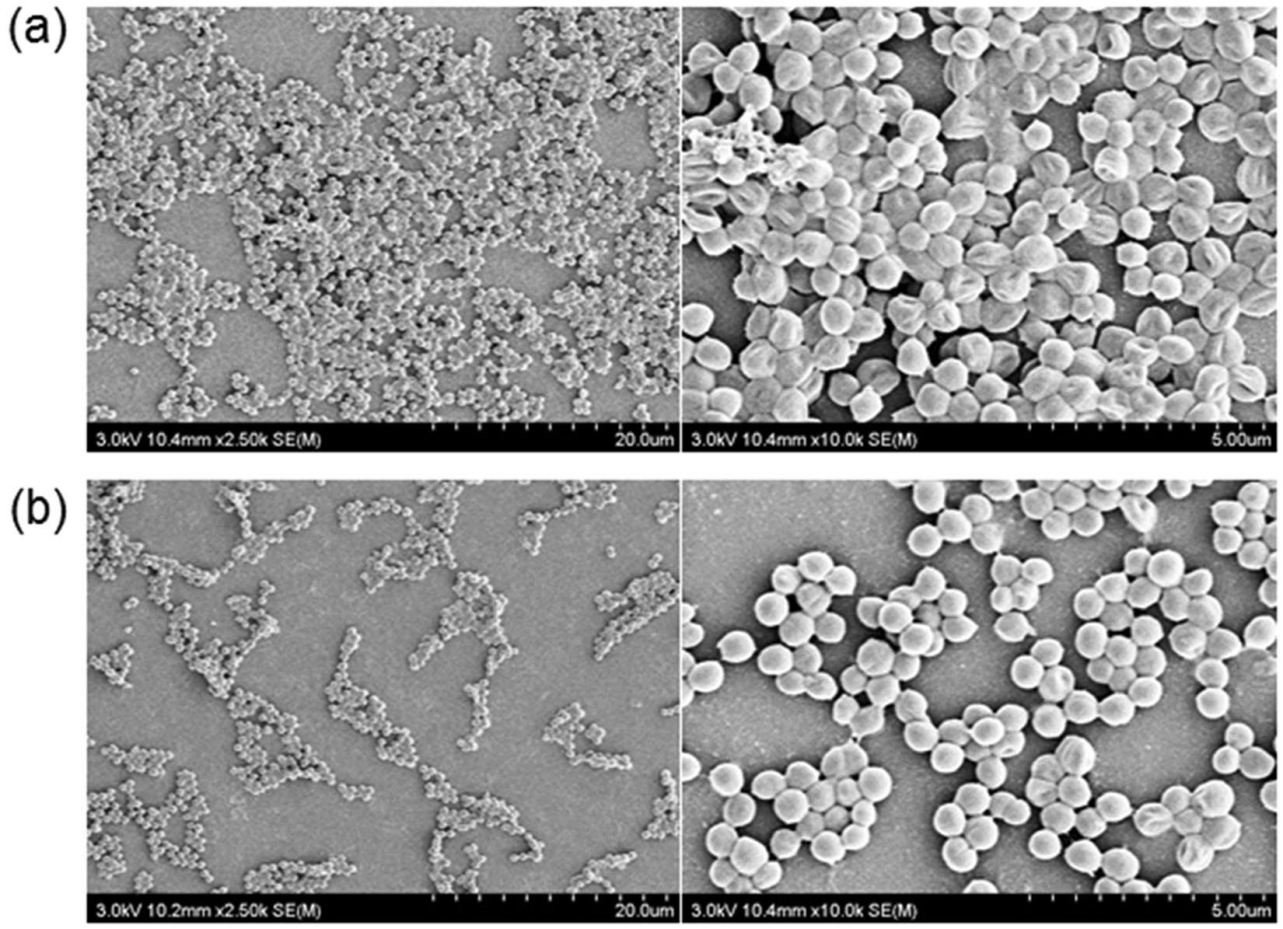
SEM images of *S. mutans* biofilms after the treatment (**a**) with DW and (**b**) with a solution containing 5.6 mM betaine and 2.8 mM erythritol.

The detachment of biofilms was directly confirmed by monitoring images of scanning electron microscope (SEM). Biofilms were immersed in DW and the betaine-erythritol solution for 10 min, respectively, and then gently washed. The betaine-erythritol solution contained 5.6 mM betaine and 2.8 mM erythritol. Namely, the mixture was contained at a concentration of 0.1% by weight. SEM images were obtained after bacterial fixation and Pt-coating processes. As shown in Fig. 3, after contact with the mixture of betaine and erythritol, approximately half of the adhered bacteria disappeared.

### Causes of biofilm detachment

In order to identify the cause of biofilm detachment, adhesive energy of biofilms was evaluated with atomic force microscope (AFM) using a functionalized AFM tip^47^. *S. mutans* biofilms were treated with a betaine-erythritol solution at a concentration of 0.1% by weight. After the treatment, adhesive energy was quantified from force-distance spectroscopy that shows retractive force of the tip from the surface. The adhesive force, which involves elastic properties, is typically obtained with conventional sharp-shaped probe tips by measuring the degree of distortion of the cantilever as the tip retracts from the surface^48^. The conventional tip, however, has a limitation that the tip does not reflect the actual force generated by the attraction from the surface because of excessive contact stress on the surface during a low-pressure load. To increase the accuracy of force-distance spectroscopy, a large contact area between the tip and the surface of substrate is required^49^. On that account, a spherical silicon oxide probe has been employed.

**Figure 4:**
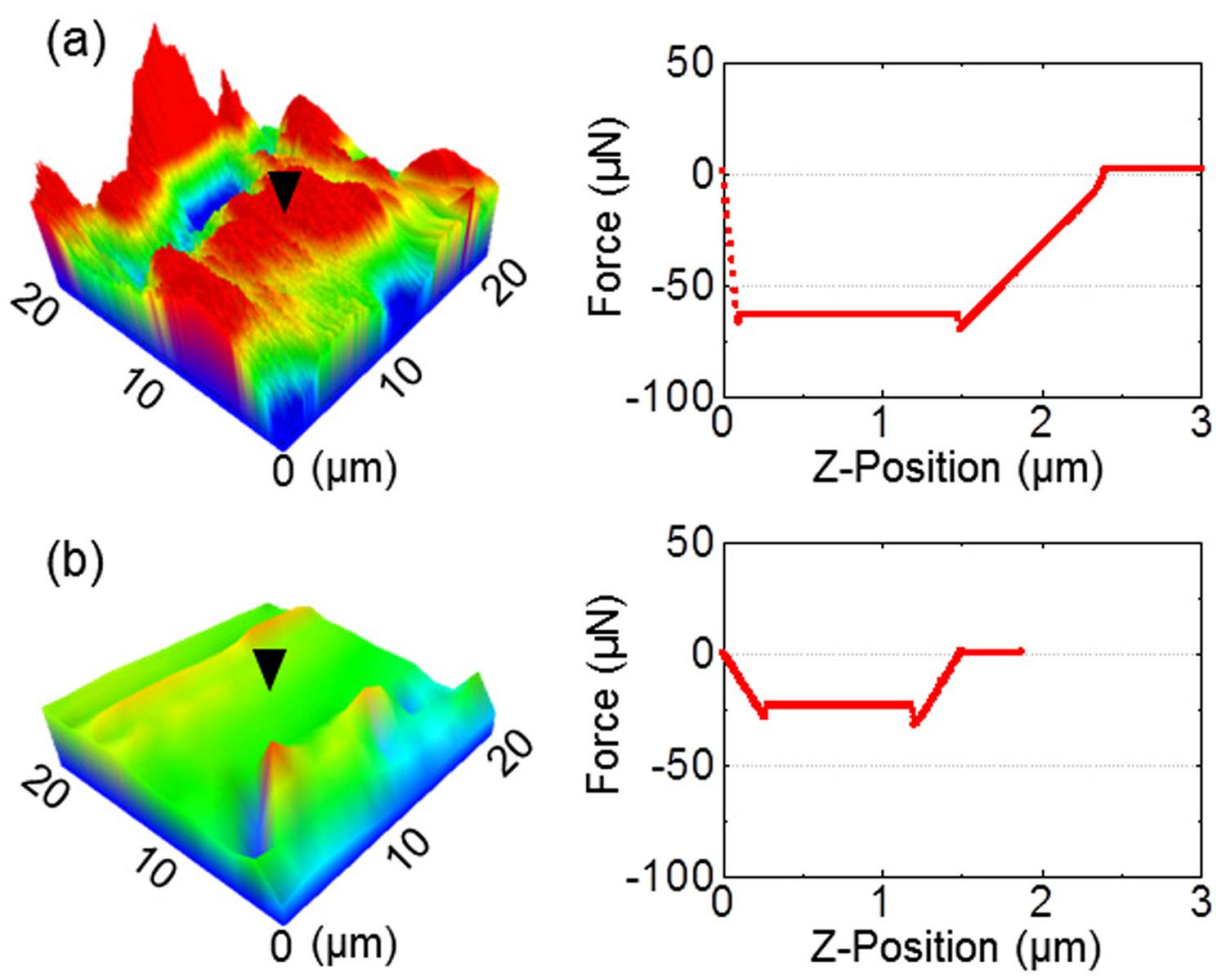
AFM images (left) of morphology of biofilm surfaces and force-distance curves (right) after the treatment with (**a**) DW and (**b**) betaine-erythritol solution. The betaine-erythritol solution contained betaine of 5.6 mM and erythritol of 2.8 mM. The inverted triangle represents where the force-distance curve was measured.

As shown in Fig. 4, after the biofilm was contacted with the betaine-erythritol solution, less adhesive strength was observed compared to that after contacted with DW. The average adhesive energy of 1.80 ± 0.01 mJ m^−2^ was obtained in the force-distance spectroscopy on the surface of biofilms contacted with the mixture, whereas the average energy of 7.11 ± 0.35 mJ m^−2^ was involved in the case of DW.

**Figure 5:**
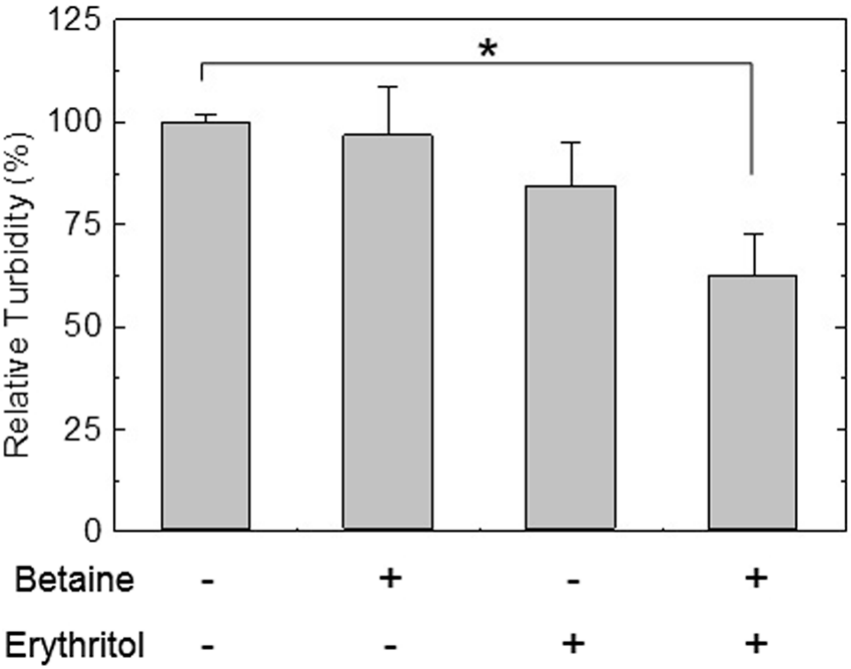
Relative turbidity of suspensions containing 1% water-insoluble polysaccharides isolated from *S. mutans* biofilms. 5.6 mM betaine and 2.8 mM erythritol were contained in DW. Each value and error bar indicates mean and SD, respectively (*n* = 3–5).

One of the most important reasons for adhesion of microorganisms is extracellular polysaccharides of biofilms^50^. Therefore, it was investigated whether exopolysaccharides originated from *S. mutans* biofilms could be dissolved in a betaine-erythritol solution to further identify the cause of the reduction in adhesive force. Water-insoluble exopolysaccharides isolated from the biofilms were suspended in the solutions containing 5.6 mM betaine, 2.8 mM erythritol and the mixture thereof, and turbidity was measured to determine polysaccharide-dissolving property. While betaine and erythritol induced slight decreases in turbidity of the suspensions, a statistically significant decrease was observed when both substances were included in the suspension (Fig. 5). The decrease in turbidity means partial dissolution of polysaccharides. And this is the result of confirming the probable cause of biofilm detachment.

## Discussion

This study provides a process to remove biofilms through a mechanism that is completely independent of general bactericidal actions. The mixture of betaine and erythritol has no antimicrobial activity. Nonetheless, the treatment with the mixture led to significant removal of biofilms by spontaneous detachment. This result is based on the assumption that betaine and erythritol could exist as a complex in water. Intermolecular interactions must rupture as water content increases, and can disappear when water is more than 50% of the total weight^51^_._ The mixture of betaine and erythritol would be indisputably separated by dilution. However, we presume that there may be also a possibility that a small fraction of undissociated complexes remains in the case of betaine and erythritol. The hydroxyl groups of erythritol have theoretically a larger dipole moment than water^52, 53^. Thus, the interaction of carboxylate in betaine with hydroxyl groups in erythritol could be preferred to that with water. Also, the anionic carboxylate of betaine has a higher hydrogen bond number than those of other zwitterions, indicating a long residence time of the coordinated molecules^54^. Due to these structural properties, it is speculated that betaine can form relatively stronger bonds with erythritol than water and that the bonds last. Actually, the residual interactions between betaine and erythritol after dilution were represented in the FT-IR and NMR spectra (Figs S1 and S2). Hence, we assume that a small amount of complexes remaining after dilution is a key factor of the synergistic effect between betaine and erythritol.

The formation of complexes composed of zwitterions and sugar alcohols can be explained by a kind of eutectic phenomenon. When betaine, erythritol and water were mixed in a ratio of 2:1:3, a clear liquid, i.e., a natural deep eutectic solvent (NaDES)^45^, was formed at room temperature (Fig. S3). The betaine-erythritol solution used in this study is not a deep eutectic solvent (DES) because betaine-erythritol mixtures are highly diluted in water. However, the formation of NaDES implies the presence of specific interactions between betaine, erythritol and water, which also supports the possibility that betaine-erythritol complexes may exist in water.

In this work, the mixture of betaine and erythritol was prepared through heating and cooling processes same as that for producing eutectic mixtures, and then diluted with water. To determine whether heating is necessary for forming interactions between carboxylate group of betaine and hydroxyl group of erythritol in water, two mixtures with or without the heating process were prepared. The position of OH peaks in NMR spectra is strongly influenced by the presence of intermolecular interactions such as hydrogen bonds^55^. Thus, if intermolecular interactions were generated by heating, a notable shift of OH peaks should be observed in the NMR spectra depending on the presence of the heating process. However, as shown in Fig. S4, no peak shift occurred, which indicates that the same intermolecular interactions exist in the two solutions. Hence, small portions of betaine and erythritol are probable to form complexes in water without heating. Furthermore, the presence of the heating did not affect the biofilm-removal efficiency (Fig. S5). From a practical point of view, the fact that a synergistic effect can occur without a heating process is very advantageous in terms of cost-saving potential. However, since it is different from eutectic phenomena, the conclusion that the synergistic effect of betaine and erythritol is due to eutectic phenomena is not clearly derived. Thus, further research is needed to understand the synergistic effect.

The efficacy of erythritol to enhance the penetration of antibiotics into biofilms has previously been proven^41, 42^. This efficacy would be caused by diffusion of low-molecular-weight sugar alcohols into EPS of biofilms^41^. Hydroxyl groups of sugar alcohols might disrupt hydrogen bonds between hydroxyl groups of exopolysaccharide which is the major component of EPS. We have assumed that this disruption by sugar alcohols could be boosted by the formation of a complex with zwitterion. This assumption was based on the hypothesis that cationic groups of zwitterion transfer the complexes to exopolysaccharides mixed with anionic components such as bacterial lipids by electrostatic interaction. The exopolysaccharides isolated from biofilms actually showed a zeta potential value of −10.9 ± 5.54 mV in deionized water (Fig. S6). Namely, the exopolysaccharides were negatively charged. And the solubility of exopolysaccharides increased by the mixture of betaine and erythritol (Fig. 5). These results are circumstantial evidence showing that intra- and inter-molecular hydrogen bonds, one of the dominant forces for the aggregation of exopolysaccharides^56^, can be disrupted in the solution containing betaine and erythritol. The formation of betaine-erythritol complex consequently led to a reduction in adhesive forces of biofilms beyond the penetration-enhancing effect.

Erythritol can also form a complex with choline chloride (ChCl) instead of betaine^45^. However, the complex of erythritol and ChCl had no effect on the removal of biofilms (Fig. S7). Choline and betaine have very similar structures, and the only difference is that their functional groups are hydroxyl and carboxylate groups, respectively. A significant difference in the formation of complexes is hydrogen bond acceptor that forms hydrogen bonds with hydroxyl groups of erythritol. In the case of ChCl-based complexes, erythritol interacts with a halide ion. The halide ion donates electron density to the quaternary ammonium group of choline and compensates for the lack of electrons at the cationic site. In contrast, in betaine-based complexes, erythritol is directly linked to the carboxylate group of betaine without the donor providing electron density to the quaternary ammonium group. The lack of electrons at the cationic site of betaine-based complexes might facilitate the transfer of the complexes to negatively-charged EPS. Hence, the direct linkage between zwitterion and sugar alcohols would be essential for the mixtures to obtain the effect of removing biofilms.

A strategy to remove biofilms with DESs, complexes comprised of organic salts and hydrogen bond donors, was proposed by M. Zakrewsky *et al*. (2014)^57^. They reported that DESs which consist of choline and carboxylic acids have antibiofilm activity. These DESs not only are an excellent agent for neutralizing pathogens inside biofilms but also have low cytotoxicity and skin irritation potential. However, the toxicological potential of choline-based DESs needs to be further validated due to their antimicrobial activity directly destroying microorganisms. In practice, the use of choline salts in cosmetics is banned by many countries including EU (Entry no 168 of Annex II to the Cosmetic Directive, EU). The mixture consisting of betaine and erythritol would offer benefits in terms of human and environmental safety. Both substances are natural food additives that are commonly ingested, and the mixture of them is safe enough that no significant decrease in viability of oral keratinocytes was observed up to a concentration of 3.3% (Fig. S8).

EPS, especially exopolysaccharides, are regarded as a critical factor promoting microbial adhesion^50^. Hence, an increase in solubility of exopolysaccharides inevitably causes a reduction in adhesive energy of biofilms and a detachment of microorganisms. However, the exopolysaccharides is not the only cause of bacterial adhesion. Thus, complete removal of the biofilms has not been achieved in this work. Further studies such as the use of zwitterion-sugar alcohol mixtures with inhibitors of bacterial adhesins^58, 59^, another major cause of microbial adhesion^60^, can be conducted to increase the biofilm-removal efficiency.

## Methods

### Preparation and analysis of samples

Betaine (Seiwa Kasei, Japan), erythritol (Ingredion Korea, Korea), glycerol, xylitol, and sorbitol (all from Daejung Chemicals, Korea) was used for the preparation of mixtures. Substances were mixed to specific molar ratios in glass vials, and heated over 90 °C until they became clear liquid states. Then, the mixtures were slowly cooled. The eutectic phenomenon was able to occur when betaine and erythritol were present in a specific molar ratio of 2:1 at high temperatures above 90 °C although the eutectic mixture of these two substances was frozen at room temperature. After the mixture became a solid, it was dissolved in water to prepare a sample. FT-IR spectra were analyzed using Avatar 320 FTIR spectrometer (Thermo Nicolet Inc., USA). The spectrometer equipped with a diamond ATR crystal accessory was used. ^1^H and 2D NOESY NMR spectra of the solution containing betaine, erythritol and H_2_O in DMSO-*d*6 were recorded at 25 °C on a 600 MHz Bruker AVANCE III spectrometer (Bruker, Germany).

### Formation of biofilm

*S. mutans* ATCC 25175 was purchased from Korean Collection for Type Cultures (KCTC). The *S. mutans* was first cultured in brain-heart infusion (BHI; Difco, USA) medium until OD_600_ reached 1.0. The cultured bacteria were diluted 100-fold in fresh BHI medium with 1% (w/w) sucrose (Daejung Chemicals). The diluted bacterial solution was transferred to 96-well plates (Nunc, Germany), and was additionally cultured in 37 °C for 16 h for the formation of biofilms.

### Assessment of biofilm-removal efficiency

After the formation of biofilms, suspended bacteria were gently removed, and sample solutions were injected to the wells. After 10 min, the sample solutions were removed using a pipette. The bacteria detached were eliminated together with sample solutions without additional washing process. After this process, fresh BHI medium with 10% (v/v) Alamar blue dye (Thermo Fisher Scientific, USA) was added to the wells. Fluorescence intensity at the 590 nm was measured using Wallac Victor3 1420 (PerkinElmer, USA) to determine the amount of residual bacteria. Removal ratios were calculated based on the fluorescence intensity of the biofilm treated with DW.

SEM images of *S. mutans* biofilms were obtained using Hitachi S-4800 (Hitachi, Japan). The biofilms were established on slide glasses for 16 h and treated with sample solutions for 10 min. After the treatment, the solutions were gently removed. Microorganisms were then fixed by phosphate-buffered saline with 2.5% (w/v) glutaraldehyde for 20 min and washed with DW. The samples were lyophilized, and coated with Pt before the acquisition of SEM images.

### Measurement of adhesive force

*S. mutans* biofilms were established on slide glasses for 16 h. The glasses were immersed in sample solutions for 10 min, respectively. Then, they were gently washed with DW, and were dried at ambient condition for 1 h. Adhesive forces were acquired in force-distance spectroscopy by AFM (XE-100, Park Systems, Korea). A spherical silicon oxide probe was employed using CP-PNP-SiO cantilever (k = 0.32 N m^−1^, NanoAndMore GMBH, Germany). A micro-sphere with a diameter of 3.5 μm was integrated on a tip-less cantilever. Since relative humidity (RH) affects adhesive force, RH was kept at 55% (±2) in a sealed chamber at 25 °C. Adhesive force was collected at five different spots per sample of 20 μm × 20 μm in force-distance spectroscopy. The force-distance curve was measured by controlling the injective length to 1 μm, and the tip was moved up to 4 μm by a pull-off motion. Adhesive energy was calculated from the curve following the tip movement. Data acquisitions were conducted at three domains of each sample, and fresh tips were used for each sample in order to avoid wrong contact stress generated by the contamination of the tip. Positions for the force-distance curve were randomly determined directly from the image by contact mode.

For monitoring topography, NCHR-10 cantilever (Nanosensors, USA) was used in tapping mode. A normal spring constant of 42 N m^−1^ was used to obtain the compliance of each tip with a typical resonance frequency of 330 kHz. Images were plane-flattened as necessary using XEI software (v. 4.3.4 build2).

### Measurement of turbidity

*S. mutans* was cultured in BHI medium with 1% (w/w) sucrose for 24 h. Suspended bacteria and medium were removed, and the established biofilm was washed with DW. After 0.5 N NaOH was injected to the biofilms, the solution was centrifuged at 10,000 × g for 30 min. The supernatant was separated and neutralized with HCl. The neutralized solution was stored at 4 °C for 48 h. Then, it was centrifuged at 10,000 × g for 30 min. The pellet fraction, water-insoluble polysaccharides, was washed five times with DW. The obtained polysaccharides were lyophilized and stored at −20 °C until use. For the measurement of turbidity, 1% (w/w) polysaccharides were suspended in sample solutions. By measuring transmission at 550 nm using Epoch microplate spectrophotometer (BioTek Instrumetns, USA), turbidity was determined. And relative turbidity was calculated based on the turbidity of a suspension containing 1% (w/w) polysaccharides.

## Conclusions

We proposed a synergistic mixture consisting of zwitterion and sugar alcohols. The mixture induced the spontaneous detachment of *S. mutans* biofilms. The detachment occurred by partial dissolution of water-insoluble exopolysaccharides surrounding the biofilms. Since exopolysaccharides are one of the major reasons for bacterial settlements, the dissolution of exopolysaccharides led to a decrease in adhesive forces of the biofilms. The biofilms, which lost adhesive forces, were consequently detached from the surface to which they had been adhered. The mixture of betaine and erythritol, which are edible substances, can be extensively utilized for the simple and safe removal of biofilms.

## Electronic supplementary material

Supplementary Information
