# Supplementary material for: Spontaneous detachment of Streptococcus mutans biofilm by synergistic effect between zwitterion and sugar alcohol
Source: Sci Rep. 2017 Aug 14;7:8107. doi: 10.1038/s41598-017-08558-x (PMC5556044; doi:10.1038/s41598-017-08558-x)
Supplement: Supplementary file 1 — Supplementary Information [file 41598_2017_8558_MOESM1_ESM.pdf]

Supplementary Information

Spontaneous detachment of *Streptococcus mutans* biofilm by synergistic effect between zwitterion and sugar alcohol

Jong Hyun Lim,* Sang-Hun Song, Hyun-Sub Park, Jeong Rae Lee, Sang-Min Lee

R&D Campus, LG Household & Health Care, Daejeon 34114, Republic of Korea

E-mail: jonghyun16@lgcare.com

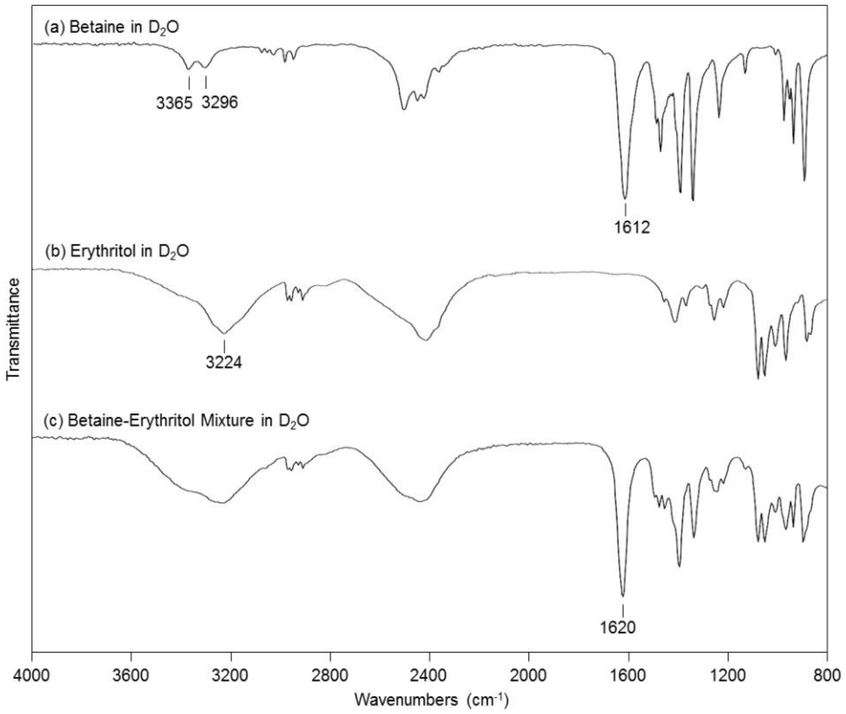

Figure S1. Fourier-transform infrared spectroscopy (FT-IR) spectra of betaine, erythritol and mixtures thereof in D_2O.

Commercially available (as-received) betaine can adsorb water molecules from atmospheric moisture[1]. The peaks at 3356 and 3296 cm^{-1} in (a) was derived from asymmetric and symmetric stretching vibration of H_2O or HDO[2,3]. And the peak at 1612 cm^{-1} in (a) was assigned to asymmetric stretching vibration of carboxylate (COO^-) in betaine[3,4]. O—D bonds were observed in the region between 2200 and 2600 cm^{-1}. In (b), the peak at 3224 cm^{-1} appeared by OH-stretching vibration and intrinsic hydrogen bonds in erythritol. When the mixture of betaine and erythritol was dissolved in D_2O, a conspicuous blue-shift from 1612 to 1620 cm^{-1} was observed as shown in (c). This shift can be explained by newly formed intermolecular hydrogen bonds between betaine and erythritol. The IR peak due to asymmetric stretching vibration of carboxylate in betaine is actually blue-shifted when the carboxylate accepts proton[4]. Thus, when betaine accepts proton by hydrogen bonding with erythritol, a blue-shift can occur. O—H bond length of erythritol is usually increased by the formation of hydrogen bonds and a red-shift of the peak at 3224 cm^{-1} should be observed[5,6]. However, the red-shift of the peak derived from hydroxyl groups of erythritol is not identified in (c). This is presumably because the peak shifted to lower wavenumbers is overlapped with the peak derived from O—H bonds that did not participate in hydrogen bonding.

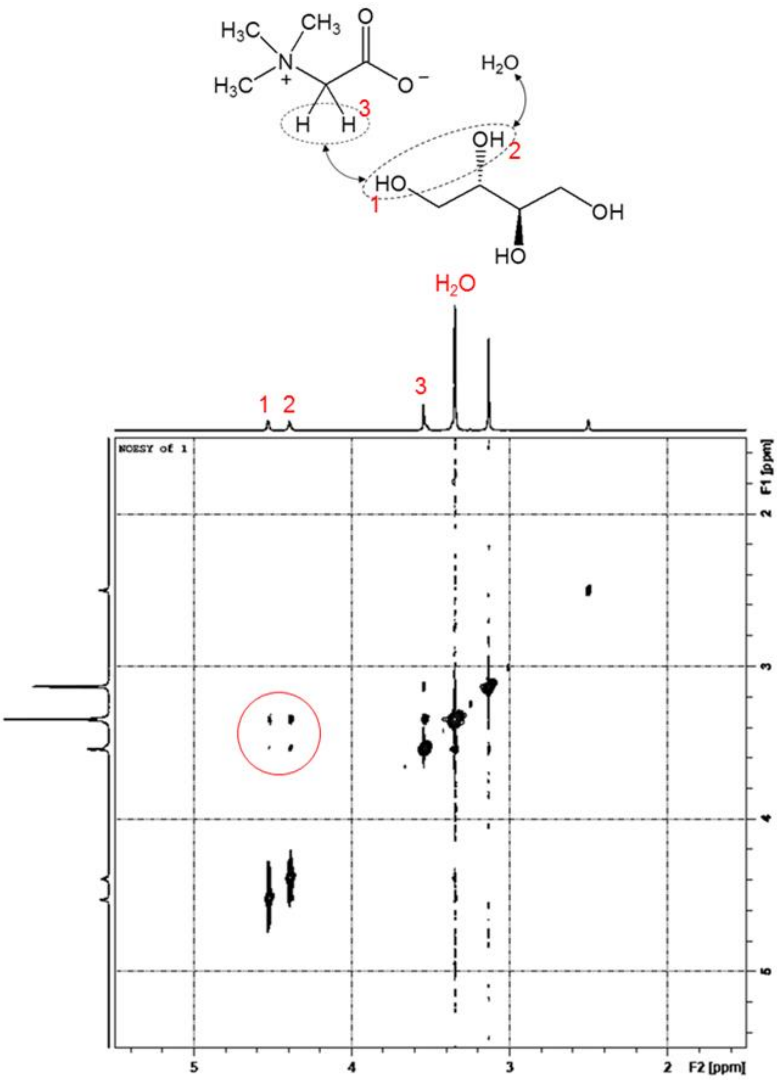

Figure S2. 2D NOESY spectrum of a mixture consisting of betaine, erythritol and H_2O with a molar ratio of 2:1:3. The mixture was completely dissolved in DMSO-*d*6 before analysis.

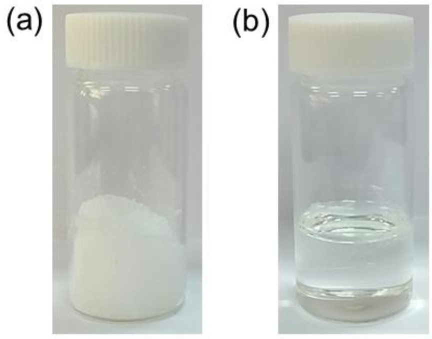

Figure S3. Mixtures of betaine, erythritol, and water with a ratio of 2:1:3 at room temperature (**a**) before and (**b**) after heated.

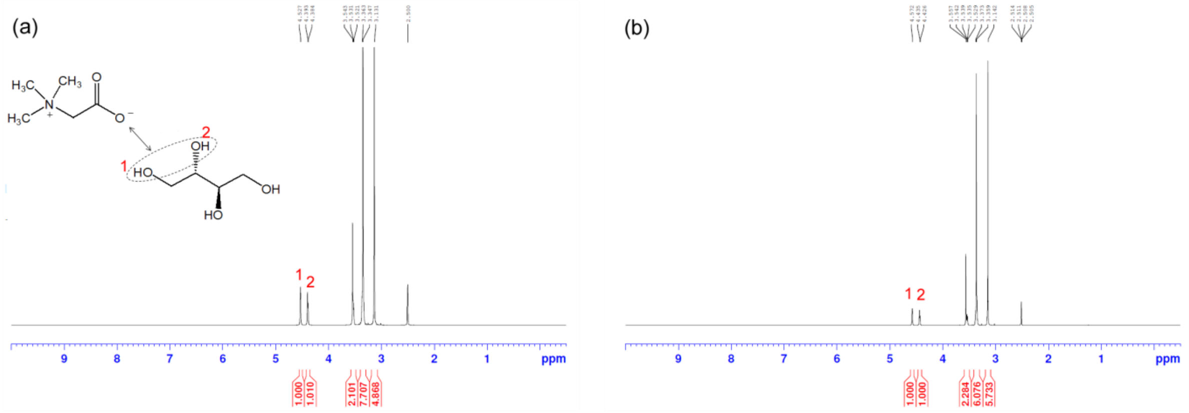

Figure S4. ^{1}H NMR spectra of mixtures composed of betaine, erythritol, and water with a ratio of 2:1:3. (**a**) Betaine and erythritol were dissolved in water and DMSO-*d*6 after heating and cooling processes. (**b**) After betaine, erythritol and water were mixed without heating, the mixture was dissolved in DMSO-*d*6. The molar ratio of betaine, erythritol and water in both samples was equal to 2:1:3, and the mixtures were diluted with DMSO-*d*6 to a final concentration of approximately 1% (w/w). No notable difference in the position of OH peaks (1 and 2) was observed.

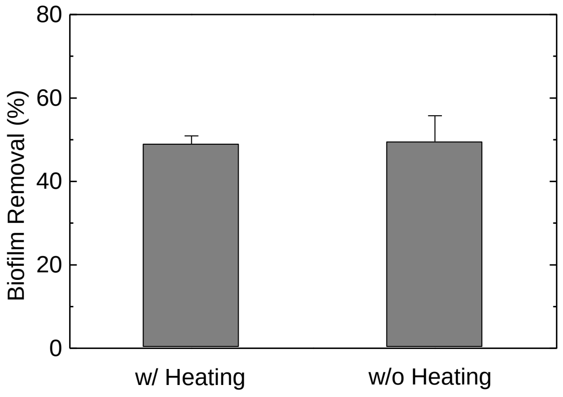

Figure S5. Effect of heating process on biofilm-removal efficiency during sample preparation. Mixtures of betaine and erythritol were heated or not heated prior to dissolution in DW. Both sample solutions contained 0.1% (w/w) betaine-erythritol mixture, and the molar ratio between betaine and erythritol was 2:1. The heating process did not affect the biofilm-removal efficiency.

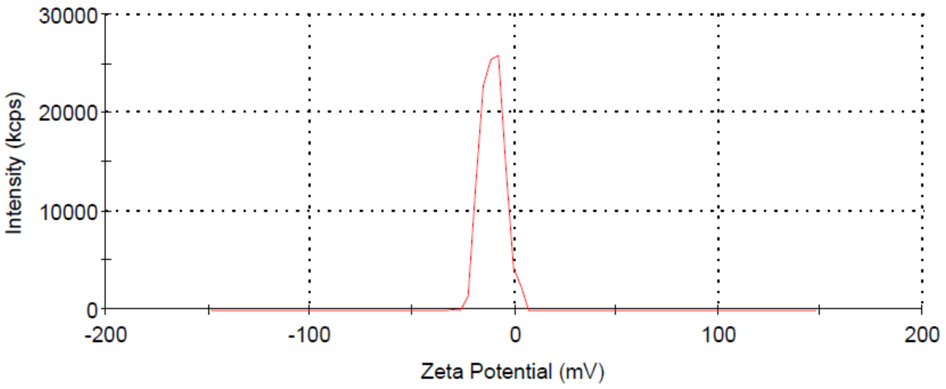

Figure S6. Zeta potential distribution of water-insoluble polysaccharides isolated from *S. mutans* biofilm. Polysaccharides were suspended in deionized water at a concentration of 0.1% (w/w). The result was obtained using Malvern zetasiser (Nano ZS, Malvern Instruments, UK).

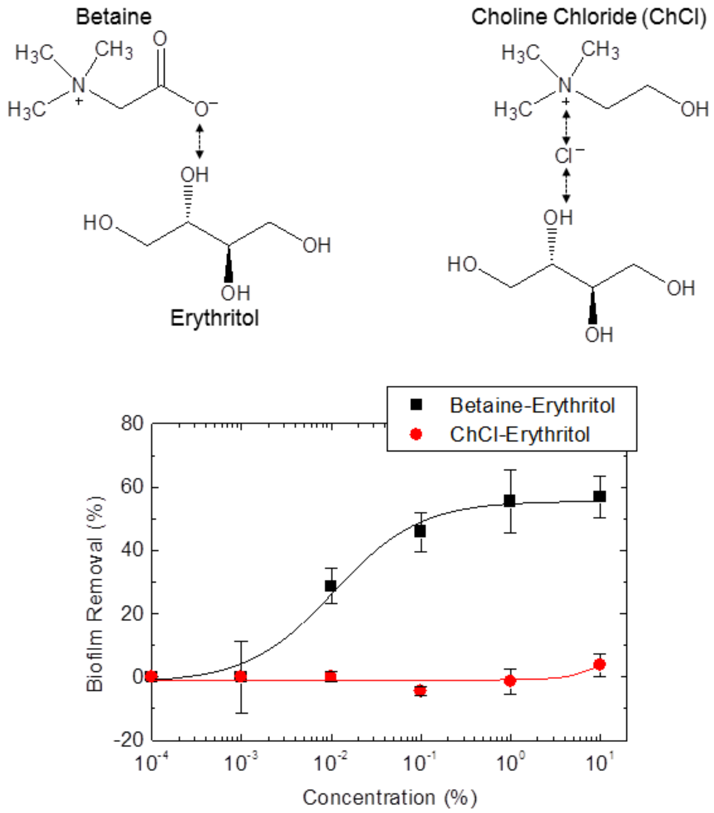

Figure S7. Comparison of biofilm-removal efficiency between betaine-erythritol and ChCl-erythritol mixtures. Betaine and erythritol were mixed at a molar ratio of 2:1. ChCl and erythritol were also mixed at the same molar ratio. Mixtures were diluted with DW.

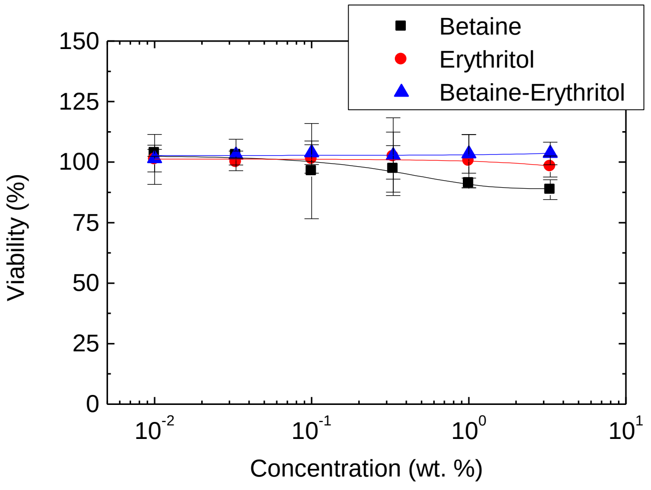

Figure S8. Viability of human oral keratinocytes after the treatment with betaine and erythritol. The betaine-erythritol mixture contained betaine and erythritol in a molar ratio of 2:1.

Human oral keratinocyte cells (#2610, ScienCell Research Laboratories, USA) were cultured in Oral Keratinocyte Medium (OKM; ScienCell Research Laboratories). The cells were transferred in 96-well plates (5000 cells/well) and incubated at 37℃ in 5% CO_2 for 24 h. Then, substances were added to the wells, and the cells were additionally incubated for 24 h. After the incubation, the culture medium was replaced with fresh medium. Cell viability was measured by Cell Counting Kit-8 (CCK-8; Dojindo, Japan) according to the manufacturer's protocol.

References

1. Toccalino, P. L., Harmon, K. M. & Harmon, J. Hydrogen bonding: Part 26. Thermodynamics of dissociation and infrared spectracrystal structure correlations for betaine monohydrate and trimethylamine oxide dihydrate. *J. Mol. Struct.* **189,** 373–382 (1988).
2. Harmon, K. M. & Avci, G. F. Hydrogen bonding: Part 15. Infrared study of hydrogen bonding in betaine hydrate and betaine hydrofluoride. *J. Mol. Struct.* **117,** 295–302 (1984).
3. Ilczyszyn, M. M., Lis, T., Baran, J. & Ratajczak, H. Structure and IR spectra of the solid complex of bis (betaine)—telluric acid. *J. Mol. Struct.* **265,** 293–310 (1992).
4. Max, J.-J. & Chapados, C. Infrared spectroscopy of aqueous carboxylic acids: comparison between different acids and their salts. *J. Phys. Chem. A* **108,** 3324–3337 (2004).
5. Guo, W. *et al.* Separation of phenol from model oils with quaternary ammonium salts *via* forming deep eutectic solvents. *Green Chem.* **15,** 226–229 (2013).
6. Dai, Y., Witkamp, G.-J., Verpoorte, R. & Choi, Y. H. Tailoring properties of natural deep eutectic solvents with water to facilitate their applications. *Food Chem.* **187,** 14–19 (2015).
